# Frequency, Risk Factors, and Outcomes of Intracranial Atherosclerotic Stenosis in Stroke Patients From the Southern Region of Saudi Arabia

**DOI:** 10.7759/cureus.43499

**Published:** 2023-08-15

**Authors:** Saeed A Alqahtani, Hanan M Abdulmutali, Haifa H Alwabel, Nawal F AbdelGhaffar, Abdullah M Ahmad, Fawziah Alahmari, Mohammad S Alqahtani

**Affiliations:** 1 Neurology, King Khalid University, Abha, SAU; 2 Neurology, Aseer Central Hospital, Abha, SAU; 3 Medicine, Kasr Al Ainy Hospital, Abha, SAU; 4 Medicine, King Khalid University, Abha, SAU; 5 Neurology, Armed Forces Hospital, Abha, SAU

**Keywords:** outcomes, risk factors, smoking, obesity, hypertension, stroke, intracranial stenosis

## Abstract

Background

Intracranial Atherosclerotic Stenosis (ICAS) represents a noteworthy cerebrovascular pathology linked to ischemic stroke, contributing to a considerable burden of morbidity and mortality on a global scale. The present study was undertaken with the primary objective of investigating the frequency, risk factors, and outcomes of ICAS in stroke patients within the Southern Region of Saudi Arabia.

Methods

This was a descriptive cross-sectional study conducted at a tertiary care hospital located in the southern region of Saudi Arabia, from June 2022 to December 2022. The study population consisted of patients aged 18 years and above who were diagnosed with acute ischemic stroke during the designated research period. Patients with hemorrhagic stroke, transient ischemic attack (TIA), or incomplete medical records were excluded from the analysis. Data pertaining to the patients were retrieved from their respective medical records.

Results

Out of 201 patients admitted with stroke, 92 (45.77%) were found to have intracranial stenosis. The majority of patients were female (52.2%) and aged over 55 years (60.9%). The presence of hypertension exhibited a statistically significant correlation with varying degrees of stenosis (p=0.02), as did ischemic heart disease and obesity (p=0.04) and active smoking (p=0.01). Hypertension displayed a marginal association with intracranial stenosis, with an odds ratio of 1.01 (95% CI: 0.25, 4.11) and a p-value of 0.02. Similarly, dyslipidemia showed a potential correlation, with an odds ratio of 1.16 (95% CI: 0.44, 3.03) and a p-value of 0.014. On the other hand, obesity showed a stronger association, with an odds ratio of 4.53 (95% CI: 1.05, 19.51) and a p-value of 0.04. Among the patients, 25 (27.17%) underwent revascularization procedures, while 44 (47.83%) were not eligible for such intervention. During the three-month follow-up, 4 (16%) experienced an ipsilateral stroke, and 3 (12%) suffered from a contralateral transient ischemic attack (TIA). Encouragingly, 18 (72%) of the treated patients showed no recurrence during the follow-up period.

Conclusion

This study concludes that approximately half (45.77%) of stroke patients had intracranial stenosis, and significant associations were found between varying degrees of stenosis and hypertension, ischemic heart disease, obesity, and active smoking. Hypertension demonstrated a marginal correlation, while obesity exhibited a stronger association with intracranial stenosis.

## Introduction

Stroke stands as a prominent contributor to mortality and morbidity on a global scale. In 2019, a total of 77.19 million people worldwide encountered an ischemic stroke, leading to 63.48 million years of life affected by disability and 3.29 million fatalities attributed to this type of stroke [1]. The primary risk factor for stroke is advancing age, and as populations age in many countries, the overall impact of stroke continues to rise [2]. In Saudi Arabia, like in many other countries, the burden of stroke cases is rising with Intracranial Atherosclerotic Stenosis (ICAS) emerging as a significant concern in this context [3]. Among the incident cases, the majority (87%) are attributed to ischemic stroke, followed by intracerebral hemorrhage (10%) and subarachnoid hemorrhage (3%) [4].

Intracranial atherosclerotic stenosis (ICAS) refers to the narrowing of intracranial arteries due to atherosclerosis, which results in reduced blood flow and an elevated risk of cerebral ischemia. The impact of ICAS on stroke outcomes can be significant and is observed to be more prevalent in specific ethnicities and regions. Various populations have shown varying prevalence rates, with Asians, Hispanics, and African Americans being more susceptible to the condition. It accounts for approximately 10% to 15% of ischemic stroke cases in Western countries [5], and its prevalence in Asia was reported to be as high as 46.6% [6]. Risk factors associated with both symptomatic and asymptomatic ICAS comprise age, Asian and black race, hypertension, diabetes mellitus, hyperlipidemia, metabolic syndrome, a sedentary lifestyle, and smoking. The mechanisms underlying stroke occurrences in ICAS encompass artery-to-artery embolization, perforator disease, and impaired distal perfusion [7].

Recent research has highlighted that the presence of intracranial atherosclerosis can worsen stroke outcomes and increase the risk of recurrent stroke [6]. Stenosis of major intracranial vessels tends to progress at a rate of 9% to 12% over six months, and individuals with more than 50% stenosis face approximately a 40% risk of experiencing a vascular event within the following two years [6].

The Southern Region of Saudi Arabia is recognized for its distinctive demographic and lifestyle factors, which may exert an influence on the epidemiology of ICAS and its consequences in stroke patients. Considerations such as genetic predispositions, dietary patterns, socioeconomic status, and access to healthcare services might play pivotal roles in determining the incidence and severity of ICAS-related strokes in this region. Through shedding light on the specific epidemiological characteristics of ICAS in this geographical area, our findings have the potential to contribute significantly to the development of targeted public health interventions and personalized treatment strategies, aimed at improving stroke prevention and management. Moreover, this research holds promise in bridging existing knowledge gaps and paving the way for further exploration of ICAS in diverse geographic and ethnic contexts.

## Materials and methods

This cross-sectional study was carried out between June 2022 and December 2022 at a tertiary care hospital located in the Southern Region of Saudi Arabia. Ethical approval for the study was obtained from the Institutional Review Board Committee, Ministry of Health Affairs-Aseer Region (REC-13-02-2022).

The research focused on adult patients aged 18 years and older, who had received a clinical diagnosis of acute ischemic stroke. This diagnosis was substantiated through a comprehensive evaluation of clinical symptoms and corroborated by neuroimaging techniques such as computed tomography (CT) or magnetic resonance imaging (MRI), all within the predetermined time frame designated for the study. Notably, patients who had experienced a hemorrhagic stroke or a transient ischemic attack (TIA), and those with incomplete or insufficient medical documentation, were intentionally excluded from the subsequent analysis. This meticulous approach in participant selection and exclusion aimed to ensure the precision and integrity of the study's findings by focusing exclusively on a homogeneous cohort of patients with confirmed acute ischemic stroke.

Trained medical personnel collected the data utilizing a standardized data collection form. Patient information was obtained from their medical records, encompassing demographic data such as age and gender, as well as risk factors like hypertension, diabetes, hyperlipidemia, smoking, and coronary artery diseases. Furthermore, data on comorbidities and the results from medical imaging procedures, such as magnetic resonance imaging (MRI), magnetic resonance angiography (MRA), and transcranial Doppler (TCD) examinations, were included in the data collection process.

Hypertension (HTN) was characterized by consistent blood pressure measurements surpassing 140/90 mmHg or the ongoing use of long-term antihypertensive medication. Diabetes mellitus (DM) was identified as fasting blood glucose levels surpassing 7.0 mmol/L, postprandial blood glucose levels surpassing 11.1 mmol/L, or the continuous necessity for hypoglycemic medications. Within the scope of this research, smoking denoted the self-reported present past smoking, while alcohol consumption referred to the self-reported ongoing or previous practice of consuming alcoholic drinks.

For data analysis, we utilized the Statistical Package of Social Science (SPSS), version 26.0 (IBM Corp., Armonk, NY). Descriptive statistics, comprising frequencies, percentages, means, and standard deviations, were computed to summarize the data. To assess associations between categorical factors and outcome variables, the Chi-squared test was employed. A P-value less than 0.05 was considered to indicate statistical significance.

## Results

Out of 201 total patients admitted with stroke, 92 (45.77%) had intracranial stenosis. Most of the patients were females 48 (52.2%) and the rest were males 44 (47.8%), and the majority 56 (60.9%) were >55 years old. Hypertension was the main risk factor in 71 (77.2%) patients, dyslipidemia in 43 (46.7%) patients, and diabetes mellitus in 35 (38.0%) patients. In the majority of the patients 50 (54.3%), the degree of stenosis was >70% (Table 1).

**Table 1:** Demographic characteristics of patients with intracranial stenosis (n=92)

Demographics	Frequency	Percentages
Age group		
15 – 25 years	09	9.8
26 – 35 years	03	3.3
36 - 45 years	09	9.8
46 – 55 years	15	16.3
>55 years	56	60.9
Gender		
Male	44	47.8
Female	48	52.2
Risk factors		
Hypertension	71	77.2
Diabetes mellitus	35	38.0
Dyslipedemia	43	46.7
Ischemic heart disease	28	30.4
Obesity	28	30.4
Smoker	20	21.7
Degree of Stenosis		
<70%	42	45.7
>70%	50	54.3

**Figure 1:**
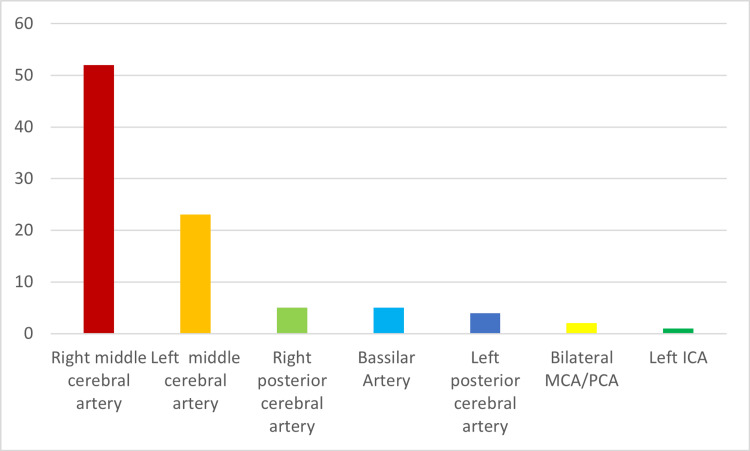
Distribution of stenosis in intracranial vessels

Figure 1 shows the distribution of stenosis in intracranial vessels. Out of the 92 cases, the majority of the stenosis was in the right middle cerebral artery (MCA) 52 (56.5%), 23 (25%) were in the left MCA, 5 (5.4%) were in the right posterior cerebral artery (PCA) and basilar artery each, and four (4.3%) were in the left PCA, with the remaining cases shown in Figure 1.

Table 2 displays the association of different risk factors with the degree of stenosis. Hypertension showed a statistically significant correlation with varying degrees of stenosis (p=0.02). Additionally, ischemic heart disease and obesity demonstrated a significant association (p=0.04), as did active smokers (p=0.01).

**Table 2:** Association of different risk factors with the degree of stenosis

Variables	Degree of stenosis		P value
	<70% (n=42)	>70% (n=50)	
Female	21 (50%)	27(54%)	0.43
Age	60.57±19.41	57.28±19.95	0.42
Hypertension	37 (8809%)	34(68%)	0.02
Diabetes mellitus	15 (35.71%)	20(40%)	0.41
Dyslipidemia	21 (50%)	22(44%)	0.35
Ischemic heart disease	17 (40.47%)	11(22%)	0.04
Obesity	17 (40.47%)	11(22%)	0.04
Smoker	6 (14.28%)	14(28%)	0.01

In this study, logistic regression was employed to identify independent factors associated with intracranial stenosis, specifically characterized by a degree of stenosis >70%. The researchers investigated several potential risk factors and their corresponding odds ratios (OR) accompanied by 95% confidence intervals (CI) to determine their statistical significance. The findings revealed that hypertension exhibited a marginal association with intracranial stenosis, displaying an odds ratio of 1.01 (95% CI: 0.25, 4.11) and a p-value of 0.02. Similarly, dyslipidemia showed a potential correlation, with an odds ratio of 1.16 (95% CI: 0.44, 3.03) and a p-value of 0.014. In contrast, obesity exhibited a stronger association, presenting an odds ratio of 4.53 (95% CI: 1.05, 19.51) and a p-value of 0.04. On the other hand, being an active smoker did not demonstrate a significant association, with an odds ratio of 1.01 (95% CI: 0.23, 4.44) and a p-value of 0.12. These findings provide valuable insights into potential risk factors for intracranial stenosis, contributing to an improved understanding and management of this condition (Table 3).

**Table 3:** Logistic regression analysis of independent markers for intracranial stenosis with >70% degree of stenosis

Variables	OR(95%CI)	P value
Male	0.65(0.21, 1.96)	0.44
Hypertension	1.01 (0.25, 4.11)	0.02
DM	1.26 (0.49, 3.25)	0.06
Dyslipidemia	1.16 (0.44, 3.03)	0.014
Ischemic heart disease	0.50 (0.18, 1.60)	0.26
Obesity	4.53 (1.05, 19.51)	0.04
Smoker	1.01 (0.23, 4.44)	0.12

Among the 92 patients, 25 (27.17%) underwent revascularization procedures, while 44 (47.83%) were found unsuitable for such intervention. Additionally, 15 (16.30%) of the patients were considered contraindicated for revascularization, and 8 (8.70%) patients or their families opted to refuse the intervention.

Among the 25 patients who underwent revascularization, follow-up revealed that 4 (16%) experienced an ipsilateral stroke, and three (12%) suffered from a contralateral transient ischemic attack (TIA) at the three-month mark. Encouragingly, 18 (72%) of the treated patients showed no recurrence during the follow-up period.

Conversely, among the 67 untreated patients, seven (10.45%) had an ipsilateral stroke, and 14 (20.90%) experienced a TIA. Unfortunately, nine (13.43%) patients were lost to follow-up. However, the majority, accounting for 37 (55.22%) of untreated patients, showed no recurrence during the follow-up period.

## Discussion

Intracranial atherosclerotic stenosis (ICAS) is a significant contributor to ischemic stroke, and understanding its frequency, risk factors, and outcomes is crucial for improving stroke management and patient outcomes. The results provide valuable insights into the epidemiological characteristics of ICAS in this specific population.

Our study findings revealed that approximately 45.77% of the stroke patients admitted to the hospital exhibited intracranial stenosis, with the right middle cerebral artery (MCA) being the most prevalent location of stenosis. This observation underscores the significance of ICAS as a commonly observed etiology of ischemic stroke in the region. A recent study conducted in Pakistan reported an intracranial stenosis prevalence of 56% among stroke patients, indicating its substantial risk factor in that specific population [8]. Similarly, a study from China identified ICAS in 32.90% of stroke patients, with its prevalence showing an increase with advancing age [9]. The prevalence of ICAS in our study closely aligns with that reported by Borhani-Haghighi et al., at 47.46% [10]. Our study's findings regarding the prevalence of ICAS-related strokes are consistent with observations from other research conducted in various Middle Eastern countries [11-13].

Moreover, the majority of the patients with ICAS were females, and the majority of them were aged over 55 years. These demographic patterns align with previous studies that have reported an increased prevalence of ICAS in older individuals and females [14]. Numerous studies have consistently demonstrated that as age advances, there is a corresponding increase in both the prevalence and severity of ICAS [15,16]. The relationship between ICAS and gender remains contentious. Several studies have demonstrated a female preponderance in ICAS [17,18].

Hypertension emerged as the most prevalent risk factor in patients with ICAS, affecting 77.2% of the cases. Hypertension is well-established as a major risk factor for atherosclerosis and stroke, and its association with ICAS in this study reaffirms its significance in the pathogenesis of this condition. Multiple clinical studies have independently established a positive correlation between hypertension and ICAS [15,19]. Dyslipidemia was also a prevalent risk factor in the study population. The association of risk factors with the degree of stenosis provided valuable insights into potential independent markers for severe ICAS. Hypertension and dyslipidemia demonstrated a statistically significant correlation with severe stenosis, suggesting that effective control of these risk factors may play a crucial role in preventing the progression of ICAS. Moreover, obesity displayed a strong link with severe stenosis, indicating the need for targeted interventions in obese stroke patients; these findings are supported by numerous studies [20-22].

Several limitations should be acknowledged to contextualize the findings appropriately. Firstly, the study's retrospective design may introduce inherent biases and limit the establishment of causal relationships between risk factors and intracranial atherosclerotic stenosis. Additionally, the reliance on medical records and data from a single region might not fully represent the broader Saudi Arabian population's diversity, potentially limiting the generalizability of the results.

## Conclusions

This study conclude that nearly half of the stroke patients had intracranial stenosis. Significant associations were found between varying degrees of stenosis and hypertension, ischemic heart disease, obesity, and active smoking. Hypertension showed a marginal correlation with intracranial stenosis, while obesity exhibited a stronger association. The findings emphasize the importance of managing and monitoring risk factors, particularly hypertension and obesity, to prevent and mitigate intracranial stenosis in stroke patients. The study highlights the need for individualized treatment plans considering the patient's condition and risk factors. The findings underscore the importance of managing these risk factors to prevent stenosis-related complications.
